# Activity-Dependent *NPAS4* Expression and the Regulation of Gene Programs Underlying Plasticity in the Central Nervous System

**DOI:** 10.1155/2013/683909

**Published:** 2013-08-18

**Authors:** José Fernando Maya-Vetencourt

**Affiliations:** ^1^Centre for Nanotechnology Innovation, Italian Institute of Technology, Piazza San Silvestro 12, 56127 Pisa, Italy; ^2^Centre for Neuroscience and Cognitive Systems, Italian Institute of Technology, Corso Bettini 31, 38068 Rovereto, Italy

## Abstract

The capability of the brain to change functionally in response to sensory experience is most active during early stages of development but it decreases later in life when major alterations of neuronal network structures no longer take place in response to experience. This view has been recently challenged by experimental strategies based on the enhancement of environmental stimulation levels, genetic manipulations, and pharmacological treatments, which all have demonstrated that the adult brain retains a degree of plasticity that allows for a rewiring of neuronal circuitries over the entire life course. A hot spot in the field of neuronal plasticity centres on gene programs that underlie plastic phenomena in adulthood. Here, I discuss the role of the recently discovered neuronal-specific and activity-dependent transcription factor NPAS4 as a critical mediator of plasticity in the nervous system. A better understanding of how modifications in the connectivity of neuronal networks occur may shed light on the treatment of pathological conditions such as brain damage or disease in adult life, some of which were once considered untreatable.

## 1. Introduction

The interaction between genetic and environmental factors lies behind the neuronal representation of sensory stimuli in the nervous system. The environment largely modifies brain structure and function through mechanisms of neuronal plasticity. Sensory experience actually drives the refinement of immature neural circuitries into organized patterns of synaptic connectivity that subserve adult brain functions [1].

Environmental influences play a key role in sculpting the central nervous system architecture during early life, when neural circuitries are highly sensitive to experience (reviewed in [2, 3]). This seems to be a period of time (so called critical period) in which an individual acquires an indelible memory of relevant stimuli in the environment, which ensures proper development of sensory functions and/or behaviours. An emerging view in the field of plasticity is that the effects caused by early developmental experience in the remodeling of neural networks seem to be actively preserved by the late appearance of structural and functional factors that restrict plasticity over the time course. This feature seems to be of relevance in terms of adaptive functions but determines diminished plasticity in the adult brain, which in turn severely restricts the functional reorganization of the nervous system thus posing a limit for feasible clinical interventions after brain injury or disease in humans (for review see [4, 5]).

The capacity of neural circuitries to change in response to sensory experience is of high relevance in fields of neuronal rehabilitation and brain repair. This is clear, for instance, in the case of stroke, which is a major cause of long-term disability for which there is currently no clinical treatment. Reactivating juvenile-like plasticity in the adult brain would be beneficial in poststroke patients, whose recovery depends on a reorganization of neuronal networks in adult life (reviewed in [6]).

How does experience modify synaptic circuitries in the brain? Experience-dependent modifications of brain functions depend, at least partially, on gene expression patterns that have evolved to meet specific environmental demands. The structure and function of the BDNF gene are a compelling example of physiological mechanisms by which experience-dependent plasticity is achieved. Since promoter areas of the BDNF gene are differentially regulated by distinct neurotransmitter systems, the levels of which vary in response to environmental influences (for review see [7]), BDNF protein synthesis in the brain is regulated by experience in a spatiotemporal-dependent manner [8, 9]. This neurotrophin drives different forms of synaptic plasticity and therefore epitomizes how the nervous system mediates fast adaptive responses to changing environmental conditions.

A hot spot in the neuroscience field is the identification of physiological mechanisms associated with experience that trigger alterations in the pattern of DNA methylation and/or posttranslational modifications of histones that in turn control the expression of genes underlying phenomena of plasticity in the brain (reviewed by [10, 11]). Indeed, epigenetic mechanisms that exert a long-lasting control of gene expression by modifying chromatin structure rather than changing the DNA sequence itself have been recognized as experience-dependent mechanisms that regulate the occurrence of brain plasticity ([12–15], reviewed in [16–18]).

Transcriptional mechanisms that are mediated by immediate early genes (IEGs) and lie behind the occurrence of plasticity in the nervous system have also been subject of recent studies ([19], for review see [20, 21]). It is becoming increasingly clear that experience-dependent plasticity is achieved when neuronal activity triggers intracellular signal pathways that promote the induction of IEGs (e.g., *c-Fos*, *c-Jun*, *CREB,* and *Zif268*) that in turn control the expression of downstream targets, the products of which then work via the activation of structural and functional mechanisms that eventually modify the strength of synaptic connections so as to change the computational properties of neural networks in the brain (reviewed in [20–22]). 

In this review, I shall focus on the role of the recently discovered neuronal-specific transcription factor NPAS4 as a key regulator of brain plasticity and cognition. It has been suggested that NPAS4 may be involved in phenomena of plasticity after local [23, 24] and global [24, 25] cerebral ischemia, seizures [26, 27], and brain injury [25, 27, 28]. More recently, it has been reported that the NPAS4 transcription factor plays a key role in mediating a transcriptional program underlying amygdala-dependent [29] and hippocampal-dependent [30] processes of memory, social, and cognitive functions [31]. The upregulation of NPAS4 in the striate nucleus after chronic amphetamine administration [32], which is a pharmacological model of plasticity with high relevance for mechanisms of addiction [33, 34], has also been described. Moreover, impairments of neurogenesis and deficits in fear [35] and spatial memories [36] by social isolation and chronic stress seem to be associated with the transcriptional suppression of the *NPAS4* gene [37], suggesting a central role for this transcription factor as a mediator of plasticity. Here, I will highlight recent advances that have brought to light some of the structural and functional mechanisms underlying the action of NPAS4 in experience-dependent plasticity. I shall also cover novel findings on NPAS4-mediated gene programs that lie behind phenomena of cortical plasticity caused by either pharmacological treatments or experimental strategies based on the enhancement of environmental stimulation levels in adult life.

## 2. Neuronal Activity and *NPAS4*-Mediated Gene Expression Patterns

Studies aimed at the identification of genes that mediate the activity-dependent regulation of inhibitory synapses formation during development, revealed that NPAS4 is an IEG induced by neuronal activity that seems to lie behind homeostatic mechanisms that keep neuronal firing in response to sensory experience within normal levels [38].

The exposure of primary neuronal cultures to high levels of potassium chloride leads to membrane depolarization and calcium influx through L-type voltage-sensitive calcium channels [39]. The resulting increase in intracellular calcium levels then triggers calcium-dependent signaling pathways that eventually mediate changes in gene transcription. The analysis of DNA microarrays upon this experimental design, in cortical neurons of young mice when development of inhibitory synapses is underway, revealed that NPAS4 is a transcription factor regulated by neuronal activity, whose expression parallels the development of inhibitory synaptic contacts [38].

*NPAS4* is selectively induced by calcium influx only in neurons but not in other cell types. The expression of *NPAS4*, unlike other activity-dependent transcription factors such as *CREB* and *c-Fos*, is triggered selectively by excitatory synaptic transmission but not by neurotrophic factors [38]. As observed in the cortex, *NPAS4* expression in primary hippocampal neurons increases with the formation and maturation of synaptic contacts that occur during development, presumably, because of enhanced endogenous spontaneous levels of activity. Of note, *NPAS4* expression in response to stimulation of primary sensory areas has been reported; visual experience in mice after one week of dark exposure actually increases mRNA and protein levels of NPAS4 in visual cortex pyramidal cells [38]. Interestingly, the expression of *NPAS4* seems to take place predominantly in excitatory neurons.

The induction of *NPAS4* promotes GABA-mediated inhibitory transmission during development. Studies in hippocampal cell cultures, using shRNA interference (shRNAi) against *NPAS4* and immunohistochemistry for both the GABA synthetizing enzyme GAD65 and the GABAA-receptor *γ*2 subunit as pre- and postsynaptic markers, respectively, revealed that the downregulation of *NPAS4* expression markedly reduces inhibitory synaptic contacts formation on perisomatic and dendritic regions of excitatory neurons, suggesting that this transcription factor positively regulates the number of inhibitory synapses that form during early life. These findings were confirmed by recordings of miniature inhibitory postsynaptic currents (mIPSCs) in CA1 pyramidal cells, which decrease in amplitude after *NPAS4* downregulation by *NPAS4*-shRNAi infection [38]. Furthermore, experiments performed in conditional knockout mice (*NPAS*4^flx/flx^) in which the *NPAS4* gene is selectively deleted by CRE-mediated recombination revealed that CRE expression leads to a significant increase of interevent intervals of mIPSCs, thus showing that CA1 pyramidal neurons lacking *NPAS4* receive fewer inhibitory synaptic inputs. In contrast, increasing *NPAS4* levels in cultured hippocampal neurons enhances the formation of inhibitory synapses, as suggested by a marked increase in the number of the GABAA-receptor *γ*2 subunits. In line with this, expression of *NPAS4* in CA1 pyramidal neurons increases the amplitude of mIPSCs while decreasing mIPSCs interevent intervals, consistent with an enhanced inhibitory synaptic signaling [38].

Notably, modifications of excitatory synaptic transmission also seem to occur after alterations of *NPAS4* expression. In addition to the induction of genes that control the development of inhibition, *NPAS4* also seems to regulate a gene program that includes a wide variety of transcription factors, genes encoding channel proteins, G-protein signaling molecules, protein kinases and phosphatases, and genes involved in membrane receptors trafficking and synaptic transmission [38]. Moreover, it has been reported that NPAS4 mediates *BDNF* expression in primary cortical neurons [30, 38, 40, 41]. *BDNF* is reduced in neurons with decreased levels of *NPAS4* after lentiviral *NPAS4*-shRNAi infection and primary cell cultures from NPAS4 knockout mice consistently show a similar reduction of depolarization-induced *BDNF* expression [38]. Chromatin immunoprecipitation (ChIP) studies have shown that NPAS4 binds to the BDNF promoters I and IV in membrane-depolarized neurons, indicating that NPAS4 directly mediates the activity-dependent *BDNF* transcription. This phenomenon seems to underlie, at least partially, the effect of NPAS4 in increasing the formation of inhibitory synapses, as the number of inhibitory synaptic contacts induced by *NPAS4* is moderately attenuated in cells in which *BDNF* has been knocked down by *BDNF*-shRNAi infection. Accordingly, the enhancement of inhibition caused by *NPAS4* in CA1 neurons is partially but not totally attenuated by knocking down *BDNF* levels [38].

In summary, *NPAS4* induction in response to excitatory transmission appears to mediate a reduction of neuronal activity levels and therefore may function as a homeostatic mechanism during phases of enhanced excitability [38]. To what extent NPAS4 mediates, directly or indirectly, the development of inhibitory synaptic contacts formed by different types of GABAergic interneurons on excitatory cells is an open question that remains to be explored. Further studies of NPAS4 physiological functions may shed light on mechanisms by which experience-dependent neuronal activity regulates the balance between inhibition and excitation in the brain and how alterations in such a balance may contribute to pathological conditions such as Down syndrome, Autism, and Rett syndrome in which inhibitory transmission seems to be altered [42–44].

## 3. NPAS4 Upregulates a Gene Program That Underlies Memory Formation

The formation and storage of memories are a classical example of experience-dependent plasticity mechanisms that allow an individual to modify behaviour by learning. What structural and functional changes occur in the brain as we learn? It is well established that there are stages in memory that are encoded as modifications in the strength of synapses that correlate with behavioural phases of short- and long-term memory.

**Figure 1:**
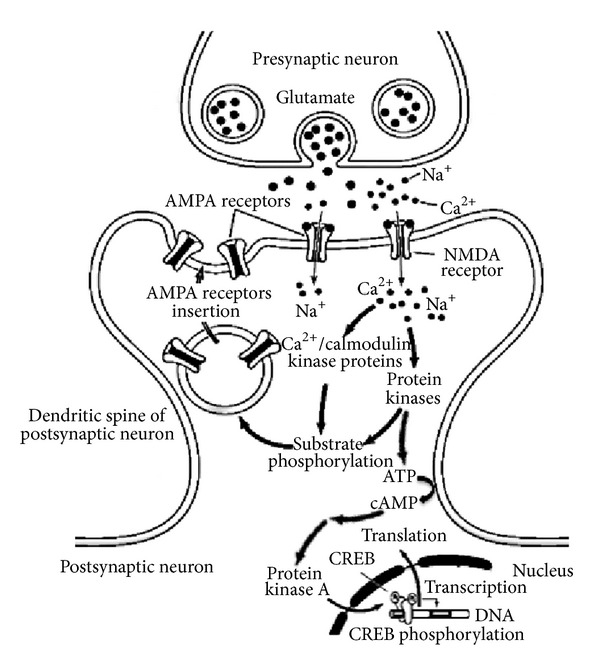
Molecular mechanisms underlying long-lasting modifications of synaptic transmission. After presynaptic glutamate release, the NMDA channel opens only when the postsynaptic neuron is sufficiently depolarized. As a result, the permeability of Ca^2+^ increases and Ca^2+^ ions activate postsynaptic protein kinases. These kinases may then act to insert new AMPA receptors into the postsynaptic spine, thereby increasing the sensitivity to glutamate. The activation of second-messenger pathways (e.g., ↑ cAMP) that subsequently set in motion the catalytic subunit of the protein kinase A results in the phosphorylation of the transcriptional regulator CREB. This turns on the expression of a number of genes (those containing the CRE promoter area) that produce long-lasting structural and functional changes on the synapses.

Pioneering studies from molluscs to flies, and mammals revealed highly conserved signal transduction pathways that are critical for the occurrence of synaptic plasticity underlying the establishment of long-term memories. These conserved pathways involve calcium-mediated activation of intracellular protein kinases, translocation of these proteins to the nucleus, and subsequent activation of transcription factors that mediate gene transcription (for review see [45]). Activation of Glutamate *N*-methyl-*D*-aspartate (NMDA)-receptors [46], for instance, seems to induce phosphorylation of CREB, which causes alterations of chromatin structure that allow for the induction of gene programs and *de novo* synthesis of proteins that eventually mediate long-term changes of synaptic transmission during learning [47] (Figure 1).

In rodents, the hippocampus is involved in the formation of memory for new environments or contexts (reviewed by [48]), this phenomenon being dependent on the activation of the CA3 hippocampal area [49–51]. Contextual memory formation can be examined using the contextual fear conditioning (CFC) task (for review see [52]), which consists of exposure of an animal to a given context in which an electric shock, that may or may not be accompanied by a tone, occurs. After training in this protocol, wild-type animals normally remember and associate the context with the aversive shock experience, which can be later evaluated in terms of freezing behaviour; 1 hour or 24 hours after training, the animals are exposed to the same aversive context to explore either short- or long-term contextual memory, respectively. Using this experimental paradigm, a novel role for NPAS4 in the regulation of contextual memory formation has been recently uncovered [30].

These studies initially evaluated the expression of the IEGs *c-Fos*, *Arc*, and *NPAS4* in the dorsal hippocampus of mice that were exposed to the CFC task and sacrificed at different time points. Notably, *NPAS4* expression was found to peak much before that of *c-Fos* and *Arc*; *NPAS4* mRNA reached its peak after 5 min of training, returning to basal levels of expression after 4.5 hours. Instead, *c-Fos* and *Arc* reached their peak levels of expression after 30 min of training [30]. These findings highlight a hierarchical genetic program in which *NPAS4* is upstream of several other IEGs in the dorsal hippocampal area. This notion was later confirmed by the observation that conditional deletion of the *NPAS4* gene by CRE recombination in hippocampal neurons of *NPAS*4^flx/flx^ transgenic mice results in a marked loss of *c-Fos*, *Arc,* and *Zif268* expression [30].

Learning and memory deficits were also evaluated in *NPAS4* knockout (*NPAS4* ^−/−^) mice. After 5 min of training in the CFC test, robust freezing behaviour was observed in both wild-type and *NPAS4* ^−/−^ littermates, indicating that learning capabilities were normal in *NPAS4* ^−/−^ animals. In contrast, freezing behaviour was significantly reduced 1 hour and 24 hours after CFC training, showing that both short-term and long-term memory formation is impaired in *NPAS4* ^−/−^ mice [30]. 

After CFC training,* NPAS4* expression was localized mainly in the CA3 area of the hippocampus. The selective deletion of *NPAS4* in CA3 but not in CA1 impaired long-term contextual memory formation; 24 hours after CFC training, *NPAS*4^flx/flx^ mice injected in CA3 with a virus expressing the CRE recombinase showed attenuated freezing responses as compared with wild-type or *NPAS*4^flx/flx^ animals injected in CA1 [30], thus demonstrating that deleting *NPAS4* specifically in CA3 replicates the memory deficits seen in the *NPAS4* knockout.

The issue of whether *NPAS4 *expression in the CA3 area of the *NPAS4* ^−/−^ background leads to the expression of the *NPAS4*-mediated gene program and rescues memory formation was also investigated. Remarkably, the expression of *NPAS4* in CA3 completely reversed the short-term and long-term contextual memory deficits previously observed in the *NPAS4* ^−/−^ background; *NPAS4*-expressing mice in CA3 but not in CA1 showed similar freezing behaviour as wild-type control animals after either 1 hour or 24 hours of training in the CFC behavioural task [30]. Consistently, the same experimental design also induced *c-Fos* expression in CA3.

In summary, this elegant set of experiments demonstrates that the activity-dependent transcription factor NPAS4 is a key mediator of plastic phenomena that underlie hippocampal-dependent contextual memory formation. On the one hand, acute deletion of the *NPAS4* gene in CA3 results in a dramatic diminishment of IEGs expression and impaired contextual memory formation. On the other hand, expression of *NPAS4* mRNA in NPAS4 knockout animals effectively restores both IEGs expression and memory formation. 

## 4. Role of NPAS4 in the Regulation of Homeostatic Plasticity

The first model to provide a specific mechanism for modifications of synaptic transmission involved in associative learning was advanced by Donald Hebb in 1949; it was proposed that modifications in the strength of synapses might occur only if the use of those synapses was associated with and contributes to the generation of action potentials in the postsynaptic neuron (reviewed by [53]). Hebb's principle has been summarized as follows: “*neurons that fire together wire together*” whereas “*neurons that fire out of synchrony lose their connection*.” Thus, an essential feature of this postulate is that modifications of synaptic transmission depend on coincidence activity of the presynaptic and the postsynaptic neuron. NMDA-receptors actually function as coincidence detectors in synaptic plasticity, as they open and mediate excitatory synaptic transmission only when the presynaptic release of glutamate is coupled to the postsynaptic depolarization ([54, 55], for review see [56]), thus fulfilling Hebb's rule at molecular level. 

Although Hebbian mechanisms provided an initial and important framework for the interpretation of neuronal network alterations, it has become clear that there are mechanisms of metaplasticity controlling changes of synaptic plasticity (reviewed by [57]). Indeed, due to positive feedback, Hebbian plasticity could lead to a saturation of the synaptic strength in the absence of proper constraints. There is now a general consensus that homeostatic mechanisms are regulatory adjustments that work to maintain the stability and functionality of neuronal networks when modifications of synaptic transmission are underway (reviewed in [58]).

A classical form of homeostatic plasticity is epitomized by the Bienenstock-Cooper-Munro (BCM) model [59], which states that synaptic inputs driving postsynaptic firing to high levels result in an increase in synaptic strength, whereas inputs that trigger low levels of postsynaptic firing result in a decrement of synaptic transmission. The threshold for neuronal activation in the BCM model is not fixed but changes itself as a function of postsynaptic activity, the threshold slides as to make potentiation more likely whenever average activity is low, and less likely when average activity is high (reviewed by [60]). This is thought to maintain the stability of synapses in neuronal circuitries upon changes of synaptic transmission.

Mechanisms of homeostatic plasticity described so far (for review see [57, 58]) include (i) synaptic scaling (i.e., scaling of the strength of excitatory synapses depending on the average activity of the postsynaptic neuron) and (ii) the regulation of intrinsic excitability (i.e., changing the way in which postsynaptic neurons integrate synaptic inputs and fire action potentials). The identification of molecular substrates underlying these forms of homeostatic plasticity, however, still needs further research. Hence, the discovery that the activity-dependent expression of *NPAS4* is implicated in a transcriptional program that regulates neuronal firing responses to excitatory transmission by enhancing inhibition [38] is of high relevance for homeostatic plasticity research. It will be interesting to evaluate mechanisms of metaplasticity in *NPAS4* ^−/−^ knockout animals or in conditional *NPAS*4^flx/flx^ mice after deletion of the NPAS4 gene by selective CRE recombination.

## 5. NPAS4 and Structural Plasticity in the Nervous System

Experience-dependent functional modifications of neuronal circuitries in the brain are accompanied by structural rearrangements of neuronal connectivity. Excitatory synaptic structures such as dendritic spines, for instance, are particularly sensitive to experience during development. A total lack of visual experience in early life (dark rearing) actually modifies spines morphology and density in the visual system, these two phenomena being partially reversible by subsequent light exposure [61]. In agreement with this notion, monocular deprivation during the critical period influences motility, turnover, number, and morphology of dendritic spines in the visual cortex [62–66]. These findings highlight a correlation between the structural remodeling of single synapses and functional modifications of neural circuitries in response to changing environmental conditions.

Does structural plasticity contribute to experience-dependent changes of neuronal connectivity? This question has been recently addressed by signal optical imaging of functional responses to visual stimulation and by longitudinal two-photon imaging experiments, showing that dendritic spine dynamics of pyramidal neurons in the mouse neocortex is maximal during early stages of development but decreases thereafter, in parallel to the decline of functional plasticity that occurs over development ([65], for review see [67]). Although most studies on structural plasticity have focused on modifications in excitatory cells, there is also evidence that structural plasticity occurs in inhibitory neurons. It has been demonstrated that GABAergic interneurons in superficial layers of the visual cortex exhibit dendritic arbor growth and remodeling in adult life [68]. Moreover, structural modifications of inhibitory synapses onto pyramidal excitatory cells seem to be a major component of plasticity in the adult mouse neocortex [69–71]. The dynamic turnover of dendritic spines on pyramidal neurons and the remodeling of interneurons dendritic arbors actually appear to be a common feature among primary sensory areas [71]. In summary, cortical plasticity seems to be associated with a structural rearrangement of excitatory connections during early development whereas structural modifications of dendritic arbors in GABAergic interneurons seem to correlate with adult cortical plasticity.

An unresolved and interesting question in the field is whether NPAS4 activates downstream targets associated with structural plasticity in the nervous system. Very recent studies suggest that NPAS4 may be involved, at least in part, in some forms of structural plasticity. There is evidence that differentiation-induced neurite outgrowth in cell cultures is inhibited if *NPAS4* expression is knocked down, whereas overexpression of *NPAS4* appears to accelerate neurite outgrowth [72]. Moreover, depolarization-induced neurite outgrowth is impaired in the hippocampus of *NPAS4* knockout animals. This phenomenon appears to depend on phosphorylation of the protein synapsin-I by the cyclin-dependent protein kinase CDK5 and NPAS4 seems to mediate *CDK5* expression by binding to the CDK5 gene promoter area [72]. Whether these findings bear any physiological significance in naturally occurring processes of neuronal plasticity is an open question to be explored. It may be interesting to examine whether dendritic spines in excitatory neurons and dendritic arbors in GABAergic cells are fewer and/or lessened in the visual cortex of *NPAS4* ^−/−^ mice or in conditional *NPAS*4^flx/flx^ mice after deletion of the NPAS4 gene by selective CRE recombination.

Since the expression of *NPAS4* seems to take place predominantly in excitatory neurons [38], another interesting consideration would be to investigate whether NPAS4 influences dendritic spines turnover and density in pyramidal cells in the visual cortex. This issue is of particular relevance for the process of plasticity reactivation late in life as there is evidence that the maturation of the extracellular matrix composition during development stabilizes neuronal connectivity patterns while inhibiting structural and functional plasticity of dendritic spines [73]. The degradation of extracellular matrix components known as chondroitin sulphate proteoglycans (CSPGs) by exogenous administration of the bacterial enzyme chondroitinase actually reinstates ocular dominance plasticity in adulthood [74], probably by modifying dendritic spines dynamics and associated neuronal connectivity changes in the visual cortex.

## 6. *NPAS4* and the Regulation of Critical Period Plasticity in the Visual System

The extent to which environmental influences modify brain structure and function has been extensively studied in the developing visual system. An experience-dependent reorganization of eye-specific inputs during early life is actually the major mechanism by which neuronal connectivity is established in the primary visual cortex.

The monocular deprivation paradigm has been a classical model to assess neuronal plasticity in the visual system. Early electrophysiological and anatomical studies in cats and monkeys revealed that short periods of sensory deprivation by unilateral eye closure during early life cause major structural and functional modifications of visual cortical circuitries. Visual cortex responsiveness markedly shifts in favour of the non deprived eye after monocular deprivation during the critical period ([75–79]). In addition, the deprived eye becomes amblyopic; its visual acuity (spatial resolution) and contrast sensitivity are severely impaired [77, 79–81].

Is the activity-dependent *NPAS4* expression involved in the occurrence of critical period plasticity? Before addressing this question, a brief overview of the developmental functional organization of the visual system should be considered. Sensory experience during early life signals the time course of the critical period by promoting the transfer of the protein Otx2 from the retina to the visual cortex, where Otx2 appears to drive the maturation of parvalbumin-positive GABAergic interneurons [82] (for review see [83, 84]). The experience-dependent developmental maturation of GABA-mediated inhibition then establishes the threshold for both the start and the end of the critical period for visual cortical plasticity [85–87]. Indeed, transgenic mice with reduced levels of intracortical inhibition due to the lack of the GABA-synthetizing enzyme GAD65 exhibit no modifications of visual cortex responsiveness after monocular deprivation in early life, whereas enhancing inhibition by exogenous administration of GABAA-receptor agonists in the knockout background rescues the impairment of plasticity [86, 87]. On the other hand, transgenic animals that show an accelerated maturation of intracortical inhibition due to BDNF overexpression in forebrain regions display a precocious development of the visual system and an accelerated end of the critical period for ocular dominance plasticity [85].

In summary, an initial threshold of inhibition triggers a sensitive period in which neuronal networks in the visual system are highly susceptible to sensory experience, whereas a second inhibitory threshold signals the end of this phase of enhanced plasticity (for review see [88]). Since the transcriptional program activated by NPAS4 enhances inhibition by promoting the expression of genes that direct the formation of inhibitory synaptic contacts [38], it emerges clearly that *NPAS4* expression is likely involved in the regulation of the critical period for visual cortex plasticity. It will be interesting to evaluate whether *NPAS4* ^−/−^ animals with reduced levels of inhibition [38] show impairments of ocular dominance plasticity in response to monocular deprivation during early development. This could be complemented by studies of plasticity in wild-type animals after *NPAS4* downregulation by selective *NPAS4*-shRNAi infection or in *NPAS*4^flx/flx^ mice with a selective deletion of the *NPAS4 *gene by CRE recombination in the developing visual cortex. Moreover, the induction of the *NPAS4*-mediated gene program by infection with an *NPAS4* expressing virus in the visual cortex of either wild-type or *NPAS4* ^−/−^ animals may be a feasible strategy to evaluate whether *NPAS4 *overexpression accelerates visual system development and the time course of the critical period for plasticity. Another interesting issue is whether the activation of the retinogeniculocortical transfer of the protein Otx2 in the visual pathway correlates with the activity-dependent expression of *NPAS4* in pyramidal neurons of the primary visual cortex.

An alternative approach to assess the impact of *NPAS4* expression in visual cortical plasticity may be rearing animals in total darkness from birth. Experiments that combine dark rearing and electrophysiology as a functional readout have demonstrated that the absence of visual inputs during development leads to a delayed maturation of the visual cortex [89]. It will be exciting to evaluate whether dark rearing decreases the expression of *NPAS4* in the visual cortex and whether the effects of dark rearing in the visual system of wild-type animals are prevented by selective *NPAS4* expression.

## 7. *NPAS4* and Visual Cortex Plasticity in Adult Life 

Converging lines of evidence attribute the decline of plasticity that occurs with age to the maturation of intracortical inhibitory circuitries [85–87]. Consistently, it is possible to restore a high degree of plasticity in adult life by reducing levels of inhibition [90]. This is in line with the observation that experimental paradigms, such as dark exposure [91–93], environmental enrichment [94–96], food restriction [97], long-term fluoxetine treatment [15, 98, 99], exogenous IGF-1 administration [100], and genetic manipulations [101, 102], all promote plasticity in adult life by shifting the intracortical inhibitory/excitatory ratio in favour of excitation. 

Recent studies in rodents [5] and cats [103] have revealed that the process of plasticity reactivation appears to be a multifactorial event that comprises the action of different structural and functional mechanisms, working in parallel or in series, the sum of which results in the activation of intracellular signal pathways regulating the expression of plasticity genes (reviewed by [18, 104]). Indeed, experience-dependent modifications of chromatin structure that control gene transcription are recruited as targets of plasticity-associated processes in adulthood [14, 15, 97, 101]. 

Does NPAS4 drive mechanisms of visual cortex plasticity in adult life? There is evidence that NPAS4 mediates the activity-dependent expression of* BDNF* [30, 38, 40, 41], a neurotrophin that has been clearly linked to multiple forms of synaptic plasticity in diverse brain areas ([105–110], for review see [111]). Of note, chronic infusion of BDNF into the visual cortex restores susceptibility to monocular deprivation in adulthood [98], whereas the impairment of BDNF-trkB signaling effectively prevents the process of plasticity reactivation caused by fluoxetine in the adult visual system [15]. These findings portray the activity-dependent NPAS4 transcription factor as an appealing candidate for the regulation of visual cortical plasticity in adulthood. The following is an overview of the potential role of NPAS4 as a mediator of plasticity induced by different noninvasive experimental approaches in the adult visual system.

### 7.1. Pharmacological and Environmental-Like Stimulation Approaches

Compelling experimental evidence for NPAS4-mediated transcriptional mechanisms that lie behind phenomena of visual cortex plasticity in adulthood has been recently obtained using the monocular deprivation paradigm and chronic treatment with fluoxetine as a pharmacological strategy for the induction of plasticity.

There is evidence that the plastic outcome caused by fluoxetine in adulthood is accompanied by increased levels of serotonin, reduced levels of GABAergic inhibition, and increased *BDNF* expression in the visual cortex [98]. More recently, it was demonstrated that the reinstatement of plasticity caused by fluoxetine is paralleled by epigenetic modifications of chromatin structure that promote gene transcription. On the one hand, an increased histone acetylation status at BDNF promoter regions occurs in concomitance with *BDNF* expression [15]. On the other, a reduction in the methylation status at the NPAS4 promoter area parallels an enhanced *NPAS4* expression after pharmacological treatment [101]. Of note, the impairment of serotonergic signaling prevents the remodeling of chromatin structure caused by the pharmacological treatment in gene promoter areas [15]. This points toward a hierarchical model in which serotonin seems to be the *primum movens* in a series of signal transduction pathways leading to epigenetic modifications of chromatin structure and subsequent expression of plasticity genes in the adult visual cortex (for review see [18]). In this context, NPAS4 is upstream *BDNF* expression and seems to direct the gene program mediating this plastic phenomenon. Electrophysiological experiments in combination with gene delivery by lentiviral infection have actually shown that *NPAS4* expression in the visual cortex of naïve animals restores susceptibility to monocular deprivation in adulthood. Consistently, *NPAS4* downregulation by *NPAS4*-shRNAi in the adult visual system effectively prevents plastic events caused by fluoxetine treatment [101].

How does *NPAS4* fit into a model of enhanced plasticity, which correlates with a decrease of inhibition in adulthood? Given that *NPAS4* expression increases the formation of inhibitory synaptic contacts [38], one might expect NPAS4 to be inversely correlated with the occurrence of plasticity; that is, *NPAS4* expression should occlude plasticity whereas* NPAS4* knockdown should enhance it. This scenario, however, seems to be unlikely as there is evidence that knocking down *NPAS4* expression by *NPAS4*-shRNAi infection in the visual cortex of naïve animals does not restore visual cortex susceptibility to monocular deprivation in adult life [101]. Instead, based on extensive data in the hippocampus, it seems reasonable to hypothesize that the activity-dependent *NPAS4* expression caused by serotonin (fluoxetine) in the adult visual cortex may turn on a transcriptional program that upregulates the expression of plasticity genes while facilitating, in parallel or in series, a functional reorganization of inhibitory circuitries that might contribute to the homeostasis of cortical excitability during this phase of enhanced plasticity [101]. This is in line with two recent observations: (i) NPAS4 interacts with a wide variety of neuronal activity-regulated gene expression enhancers and promoters in the nervous system [112] and (ii) different homeostatic mechanisms assist to keep neuronal activity within normal levels as synaptic modifications of neuronal circuitries are underway in the visual cortex [113].

**Figure 2:**
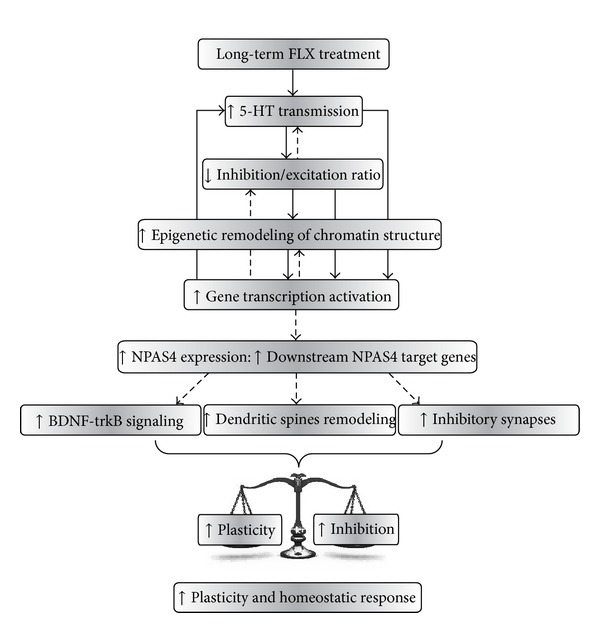
The process of plasticity reactivation in the adult visual system. The reinstatement of plasticity caused by FLX in adult life is associated with signal transduction pathways that involve the activation of long-distance serotonergic transmission, a downregulation of local intracortical inhibitory circuitries and enhanced NPAS4 expression. The experimental evidence is consistent with a model in which the increased serotonergic signaling shifts the inhibitory/excitatory balance, thus activating intracellular mechanisms that eventually promote epigenetic modifications of chromatin structure that, in turn, allow for the expression of plasticity genes in adult life, among which NPAS4 plays a key role. NPAS4 seems to turn on a transcriptional program that underlies structural and functional plasticity while facilitating, in parallel or in series, a reorganization of inhibitory circuitries that might contribute to the homeostasis of cortical excitability by driving inhibition during this phase of enhanced plasticity. The transitory expression of NPAS4 target genes may ultimately set in motion downstream physiological mechanisms that provide a permissive environment for changes in adult visual cortical circuitries (e.g., enhanced Bdnf-trkB signaling, removal of extracellular matrix components that are inhibitory for plasticity, and enhanced dendritic spines density and remodeling). Continuous arrows represent established interactions between molecular and cellular processes mentioned (boxes). Dashed lines represent interactions that remain to be ascertained. Reproduced from [101] with permission.

The available experimental evidence for a combined action of fluoxetine-induced serotonergic signaling, reduced levels of inhibition, and *NPAS4* expression in driving adult visual cortex plasticity is consistent with a model (Figure 2) in which the serotonin-mediated shift of the intracortical inhibitory/excitatory balance that occurs in favour of excitation [15, 98] may induce the activity-dependent expression of *NPAS4* [101]. This in turn could mediate the expression of plasticity genes and subsequently promote the formation of inhibitory synaptic contacts on excitatory neurons as a compensatory mechanism for the reduction of the inhibitory tone after fluoxetine treatment. In line with this notion, there is evidence that the fluoxetine-induced mechanism of disinhibition in the visual cortex of adult animals after a brief period of monocular deprivation is accompanied by an increase in elongations of GABAergic interneuron dendritic branch tips in superficial cortical layers [99]. Hence, this points toward a compensatory mechanism for the reduction of inhibition that involves the formation and/or strengthening of inhibitory contacts on neighbouring excitatory neurons, that is, a mechanism in which NPAS4 is likely to be involved.

Understanding how *NPAS4* expression regulates inhibitory synapse density and function in the adult visual system is important to interpret these findings. It may be interesting to evaluate the time course of expression of GABAergic markers in the adult visual cortex (e.g., GAD65, GAD67, VGAT, GABAA-receptor *γ*2 subunits, and parvalbumin) by means of immunohistochemistry in a naïve background versus a background of *NPAS4* knockdown or *NPAS*4^flx/flx^ mice with a selective deletion of the *NPAS4 *gene by CRE recombination. This could also be assessed in a naïve background before and after long-term fluoxetine treatment and may be complemented by electrophysiological analysis of spontaneous and evoked IPSCs to examine functional connectivity. The role of monocular deprivation in this experimental design should also be examined. The use of DNA microarrays in this context might be of relevance for the identification of NPAS4 downstream targets associated with structural and functional modifications in the adult visual system after fluoxetine treatment [114].

The enhancement of environmental stimulation levels has recently proved to be a powerful and noninvasive strategy to promote juvenile-like plasticity in the adult visual system. Environmental enrichment (EE) is an experimental paradigm characterized by enhanced sensory-motor activity and social stimulation that has a profound impact on brain structure and function (reviewed by [115]). In rodents, it has been demonstrated that short period of EE in adult life reactivates ocular dominance plasticity [94, 116] and there is evidence that resetting adult visual cortex circuitries to a more plastic stage by EE favours the rescue of sensory functions after long-term deprivation [95, 96, 117, 118]. In humans, enriching the environment in terms of body massage triggers plastic phenomena that accelerate the maturation of visual functions during development [119].

Does *NPAS4* play a role in the effects caused by EE in visual cortical plasticity? This is a likely scenario as the reinstatement of plasticity caused by EE in adulthood is accompanied by an increment in serotonin signaling, reduced levels of inhibition, and enhanced *BDNF* expression [94], much as in the case of long-term fluoxetine treatment.

It is worth noting that decreasing BDNF signaling by exogenous administration of antisense oligonucleotides in the visual cortex of adult animals exposed to enriched environmental conditions prevents partially but not totally the shift of ocular dominance in response to monocular deprivation. Considering that NPAS4 directly promotes *BDNF *expression, this suggests that the effects caused by EE in visual cortical plasticity could be only partially dependent on *NPAS4* expression. Another possibility is that NPAS4 drives phenomena of plasticity even in the absence of BDNF signaling and therefore could lie behind the plasticizing effects of EE in adult life. This is, however, an open question that remains to be explored. It will be interesting to assess the effects caused by EE in adult visual cortex plasticity in *NPAS4* ^−/−^ knockout animals or in *NPAS*4^flx/flx^ mice with a selective deletion of the *NPAS4 *gene by CRE recombination.

Brief periods of visual deprivation by dark exposure and food restriction have also proved to be effective approaches to reactivate plasticity in the adult visual system. Juvenile-like ocular dominance plasticity can actually be restored in adult animals if monocular deprivation is preceded by visual deprivation [93] or by a reduction of the caloric intake [97]. It may be interesting to investigate the effects caused by brief periods of dark exposure and food restriction in the *NPAS4* ^−/−^ knockout versus a naïve background.

## 8. Conclusion and Implications

Long regarded as a rather static and unchanging structure, the adult brain has increasingly been recognized as a system that retains a degree of plasticity that allows for structural and functional modifications of neuronal networks if exposed to certain experimental conditions. In animal models, it has been demonstrated that both pharmacological treatments and experimental paradigms based upon manipulation of environmental stimulation levels effectively promote a rewiring of visual cortical circuitries in adulthood. It seems reasonable to speculate that these noninvasive approaches, when combined with appropriate instructive environmental stimuli, could be exploited for clinical applications in humans. Since the decline of plasticity that occurs over the life course severely restricts the functional reorganization of neuronal circuitries, these studies are beginning to elucidate physiological processes that lie behind modifications of neuronal networks' connectivity in adulthood in which the activity-dependent transcription factor NPAS4 seems to play a critical role. In light of this, structural and functional mechanisms leading to the activity-dependent *NPAS4* expression arise as potential therapeutic targets for future development of drugs that could be used in a variety of pathological conditions in which a reorganization of neuronal circuitries is needed late in life. Long-term fluoxetine administration, indeed, not only induces *NPAS4* expression [101], but also has been proved successful in promoting the functional recovery from stroke in humans [120], which is a major cause of long-term disability for which there is currently no clinical treatment.
